# Supplementary material for: Inhibiting Intracellular α2C-Adrenoceptor Surface Translocation Using Decoy Peptides: Identification of an Essential Role of the C-Terminus in Receptor Trafficking
Source: Int J Mol Sci. 2023 Dec 16;24(24):17558. doi: 10.3390/ijms242417558 (PMC10744278; doi:10.3390/ijms242417558)
Supplement: Supplementary file 1 [file ijms-24-17558-s001.zip › ijms-2742503-supplementary.pdf]

Supplementary Material for:

"Inhibiting intracellular α_{2C}-adrenoceptor surface translocation using decoy peptides: identification of an essential role of the C-terminus in receptor trafficking"

Raza et al.

Tables with raw numbers for:

Figure 5: (Transient co-transfections of wild-type α_{2A}-adrenoceptors and $\alpha_{2A\text{-}2C}$-AR chimera)
Figure 6: (Transient co-transfections of wild-type α_{2C}-adrenoceptor and $\alpha_{2C\text{-}2A}$-AR chimera)
Figure 9: (Peptide specificity to inhibit receptor translocation; Peptide A and Peptide C)
Figure 10: (Human microvascular smooth muscle cells Peptide C delivery and effect on endogenous α_{2C}-adrenoceptors).

Figure 5

NIH3T3 Co-transfections
Date: August 24, 2023
Texas Red
DAPI

HA-α2A-AR-pcDNA

	BKG			Texas Red/Red Channel-BKG AVG		Corrected
#1	0.42	Cell#1-1			22.22	21.85
	0.13				21.02	20.65
	0.89				27.2	26.83
	0.28				24.71	24.34
	0.22				22.83	22.46
	0.25				23.03	22.66
AVG	**0.365**	Cell#1-2				
	**0.37**				29.54	29.17
					22.35	21.98
					28.78	28.41
					25.44	25.07
					23.44	23.07
					37.01	36.64
		Cell#1-3				
					25.81	25.44
					24.03	23.66
				tip=31.06		30.69
					30.04	29.67
					26.34	25.97
					23.33	22.96
#4	0.26	Cell#4			15.27	15.09
	0.07				13.2	13.02
	0.27			tip=18.87		18.69
	0.26				14.67	14.49
	0.03			tip=19.22		19.04
	0.19				12.89	12.71
AVG	**0.18**					
#5	0.42	Cell#5			18.18	17.97
	0.17				18.14	17.93
	0.05				15.71	15.5
	0.21				16.25	16.04
	0.23				18.74	18.53
	0.2				16.91	16.7
AVG	**0.213333**					
#6	0.53	Cell#6			23.61	23.31
	0.31				25.09	24.79
	0.33				24.55	24.25
	0.14				26.98	26.68
	0.1				25.28	24.98
	0.38				26.77	26.47
AVG	**0.298333**		**0.3**			
#8	0.09	Cell#8			34.49	34.41
	0.07				44.12	44.04
	0.09				42.05	41.97
	0.05				30.92	30.84
	0.04				29.26	29.18
	0.14			tip=21.93		21.85
AVG	**0.08**					
#12	0.39	Cell#12			26.05	25.59
	0.51				23.61	23.15
	0.68			tip=38.92		38.46
	0.52			tip=43.38		42.92
	0.48				24.79	24.33
	0.15			tip=38.13		37.67
AVG	**0.455**		**0.46**			
#13	0.02	Cell#13			23.95	23.83
	0.16				30.74	30.62
	0.12				32.12	32
	0.17				31.41	31.29
	0.21				27.52	27.4
	0.03				30.17	30.05
AVG	**0.118333**		**0.12**			
#15	0.11	Cell#15			34.63	34.52
	0.14				33.42	33.31
	0.05				30.92	30.81
	0.24				41.07	40.96
	0.04				29.38	29.27
	0.08				29.4	29.29
AVG	**0.11**					
#16	2.02	Cell#16			35.09	34.52
	0.41				40.85	40.28
	0.07				36.85	36.28
	0.45				29.77	29.2
	0.26				21.3	20.73
	0.19				30.44	29.87
AVG	**0.566667**		**0.57**			
#17	0.22	Cell#17			32.96	32.69
	0.32				40.4	40.13
	0.8				46.37	46.1
	0.16				36.05	35.78
	0.03				31.63	31.36
	0.09				36.18	35.91
AVG	**0.27**					
#18	0.31	Cell#18-1			22.7	22.47
	0.39				25.71	25.48
	0.1				29.23	29
	0.27				20.1	19.87
	0.17				17.43	17.2
	0.12				27.28	27.05
AVG	**0.226667**		**0.23**			
		Cell#18-2			21.26	21.03
					24.26	24.03
					24.54	24.31
					19.03	18.8
					19.96	19.73
					16.61	16.38
#19	0.02	Cell#19			38.99	38.61
	2.04				40.35	39.97
	0.1				36.75	36.37
	0.01				39.28	38.9
	0.03				42.96	42.58
	0.06				29.34	28.96
AVG	**0.376667**		**0.38**			**27.54511111**
						**n=15 cells**

HA-α2A-AR-Rap1A-CA

	BKG			Texas Red/Red Channel-BKG AVG		Corrected
#1	0.07	Cell#1			38.17	38.104
	0.1				44.52	44.454
	0.005				41.7	41.634
	0.06				45.53	45.464
	0.07				43.31	43.244
	0.09				45.9	45.834
AVG	**0.0658333**		**0.066**			
#3	0.36	Cell#3			16.7	16.51
	0.04				15.27	15.08
	0.005				16.29	16.1
	0.02				14.29	14.1
	0.68				17.2	17.01
	0.02				14.38	14.19
AVG	**0.1875**		**0.19**			
#4	0.1	Cell#4			10.66	10.62
	0.03				9.78	9.74
	0.04				6.79	6.75
	0.01				7.65	7.61
	0.05			tip=7.85		7.81
	0.01				8.95	8.91
AVG	**0.04**					
#5	0.06	Cell#5-1			15.82	15.75
	0.06			tip=18.76		18.69
	0.03				14.23	14.16
	0.1				14.38	14.31
	0.09				10.99	10.92
	0.07				13.67	13.6
AVG	**0.0683333**		**0.07**			
		Cell#5-2			18.85	18.78
					17.9	17.83
					22.56	22.49
					30	29.93
					22.64	22.57
					22.73	22.66
#6	0.11	Cell#6		tip=9.51		9.45
	0.06				14.88	14.82
	0.09				16.33	16.27
	0.07				19.8	19.74
	0.02				17.09	17.03
	0.02				14.85	14.79
AVG	**0.0616667**		**0.06**			**19.91538889**
						**n=6 cells**

HA-α2A-2C-pcDNA

	BKG			Texas Red/Red Channel-BKG AVG		Corrected	
#1	0.05	Cell#1			10.82	10.71	
	0.13				15.72	15.61	
	0.16				20.07	19.96	
	0.03				14.4	14.29	
	0.08				13.03	12.92	
	0.21				11.14	11.03	
AVG	**0.11**				9.25	9.14	
					10.09	9.98	
#2	0.01	Cell#2			11.18	11.04	
	0.07				9.41	9.27	
	0.27				8.06	7.92	
	0.09				7.42	7.28	
	0.24				9.6	9.46	
	0.13				8.81	8.67	
AVG	**0.135**		**0.14**				
#4	0.5	Cell#4			12.27	11.55	
	0.37				11.65	10.93	
	0.59				7.4	6.68	
	0.7				12.6	11.88	
	1.36				13.14	12.42	
	0.77				14.21	13.49	
AVG	**0.715**		**0.72**				
#5	0.33	Cell#5			18.92	18.4	
	0.45				20.52	20	
	0.33				21.34	20.82	
	0.68				16.8	16.28	
	0.95				17.8	17.28	
	0.37				10.85	10.33	
AVG	**0.518333**		**0.52**		24.19	23.67	
#7	0.35	Cell#7			9.75	8.98	
	1.13				11.57	10.8	
	0.9				13.06	12.29	
	0.96				12.8	12.03	
	0.58			tip=30.48		29.71	
	0.71			tip=14.95		14.18	
AVG	**0.771667**						
#11	0.28	Cell#11			10.57	10.13	
	0.37				8.27	7.83	
	0.33				7.96	7.52	
	0.49				9.27	8.83	
	0.68				14.18	13.74	
	0.5				14.58	14.14	
AVG	**0.441667**				8.56	8.12	
					9.61	9.17	
#13	0.21	Cell#13-1			11.03	10.36	
	0.43				12.08	11.41	
	2.22				11.36	10.69	
	0.53				13.83	13.16	
	0.5				18.65	17.98	
	0.13				18.26	17.59	
AVG	**0.67**				11.42	10.75	
					8.85	8.18	
		Cell#13-2			10	9.33	
					10.78	10.11	
					12.32	11.65	
					12.57	11.9	
					12.42	11.75	
					8.93	8.26	
					14.41	13.74	
							**12.41678571**
#15	0.14	Cell#15			10.12	9.87	**n=8 cells**
	0.27				13.57	13.32	
	0.19				14.54	14.29	
	0.16				9.96	9.71	
	0.43				11.95	11.7	
	0.29				13.83	13.58	
AVG	**0.246667**		**0.25**		10.81	10.56	
				tip=36.57		36.32	
#17	0.17	Cell#17			7.39	7.17	
	0.18				6.89	6.67	
	0.14				15.27	15.05	
	0.5				12.12	11.9	
	0.27				12.75	12.53	
	0.08				15.43	15.21	
AVG	**0.223333**				12.56	12.34	
#18	0.16	Cell#18			6.82	6.54	
	0.32				7.61	7.33	
	0.58				4.12	3.84	
	0.09				8.33	8.05	
	0.1				7.73	7.45	
	0.43				4.49	4.21	
AVG	**0.28**			tip=21.16		20.88	
				tip=13.91		13.63	
#21	0.73	Cell#21			14.52	14.12	
	0.28				14.3	13.9	
	0.05				19.74	19.34	
	0.31				11.52	11.12	
	0.53				12.99	12.59	
	0.52				13.23	12.83	
AVG	**0.403333**						
#23	0.04	Cell#23			19.97	19.91	
	0.01				18.27	18.21	
	0.07				15.86	15.8	
	0.1				18.21	18.15	
	0.1				11.1	11.04	
	0.05				14.1	14.04	
AVG	**0.061667**				10.85	10.79	
						**12.60141304**	
						**n=13 cells**	

HA-α2A-2C-Rap1A-CA

	BKG			Texas Red/Red Channel-BKG AVG		Corrected
#1	0	Cell#1		tip=49.65		49.65
	0				28.98	28.98
	0				38.29	38.29
	0				31.57	31.57
	0				41.81	41.81
AVG	**0**			tip=38.22		38.22
					30.99	30.99
#5	0.61	Cell#5		tip=34.6		34.23
	0.2				18.02	34.23
	0.41				13.59	13.22
	0.19				27.68	27.31
	0.21				25.33	24.96
	0.61				22.47	22.1
AVG	**0.371667**				24.09	23.72
#6	0	Cell#6			58.09	58.09
	0				59.96	59.96
	0				76.15	76.15
	0				85.08	85.08
	0				93.98	93.98
	0				81.64	81.64
AVG	**0**				82.05	82.05
					54.92	54.92
#7	3.07	Cell#7			33.77	32.73
	0.39				36.81	35.77
	1.03			tip=56.11		55.07
	0.43				44.41	43.37
	0.54				36.88	35.84
	0.77			tip=60.2		59.16
AVG	**1.038333**		**1.04**			
#9	0	Cell#9			40.43	40.43
	0				53.7	53.7
	0				47.85	47.85
	0				54.17	54.17
	0				47.17	47.17
	0				44.11	44.11
AVG	**0**				50.14	50.14
#12	0.2	Cell#12			61.98	61.72
	0.22				63.6	63.34
	0.41				52.37	52.11
	0.47				57.38	57.12
	0.24				39.45	39.19
	0.04				47.18	46.92
AVG	**0.263333**					
#13	2.1	Cell#13			71.43	70.47
	0.98				76	75.04
	1.07				78.81	77.85
	0.42				71.82	70.86
	0.35				59.73	58.77
	0.84				61.44	60.48
AVG	**0.96**					
#16	0.02	Cell#16			33.24	33.24
	0				62.15	62.15
	0				56.82	56.82
	0				59.95	59.95
	0				51.2	51.2
	0				38.44	38.44
AVG	**0.003333**		**0**			
#15	0.2	Cell#15			31.35	30.82
	0.54				35.43	34.9
	0.64				52.22	51.69
	0.52				60.27	59.74
	0.14				43.67	43.14
	1.11				41.01	40.48
AVG	**0.525**		**0.53**			
#10	0.01	Cell#10		tip=56.91		56.88
	0.03			tip=87.78		87.75
	0.04				22.38	22.35
	0.005			tip=50.47		50.44
	0.04				63.06	63.03
	0.05				24.68	24.65
AVG	**0.029167**		**0.03**			**49.72615385**
						**n=10 cells**

Figure 6

NIH3T3 Co-transfections
Date: Feb 23, 2023
Texas Red
DAPI
60X oil lens

mean intensity cell boundary

HA-α2C-AR/pcDNA

	**BKG**		**Red Channel- BKG AVG**		**Corrected**
#1	1.92	Cell #1-1	24.73-2.18		22.55
	1.48		11.97-2.18		9.79
	1.57		21.13-2.18		18.95
	3.39		13.02-2.18		10.84
	3.24		22.75-2.18		20.57
	1.46		24.12-2.18		21.94
AVG	**2.176666667**				
	**2.18**	Cell#1-2	19.49-2.18		17.31
			20.61-2.18		18.43
			26.49-2.18		24.31
			28.69-2.18		26.51
			19.48-2.18		17.3
			46.48-2.18		44.3
		Cell#1-3		24.91	22.73
				15.27	13.09
				16.43	14.25
				25.76	23.58
				29.93	27.75
				19.61	17.43
		Cell#1-4		16.99	14.81
				19.34	17.16
				15.19	13.01
				21.89	19.71
				15.69	13.51
				25.92	23.74
#2	3.33	Cell#2-1		24.63	22.25
	1.43			19.44	17.06
	4.72			19.56	17.18
	2.22			21.41	19.03
	1.24			30.65	28.27
	1.31			23.75	21.37
AVG	**2.375**			37.63	35.25
	**2.38**				
		Cell#2-2		29.31	26.93
				18.53	16.15
				18.11	15.73
				23.15	20.77
				35.37	32.99
				26.83	24.45
#3	2.07	Cell#3-1		25.52	23.43
	2.13			21.44	19.35
	2.07			12.68	10.59
	2.18			74.58	72.49
	1.89			19.67	17.58
	2.19			18.37	16.28
AVG	**2.088333333**			34.12	32.03
	**2.09**				
		Cell#4-1		30.98	27.53
#4	2.84			32.79	29.34
	2.74			34.49	31.04
	3.73			33.59	30.14
	2.45			62.18	58.73
	6.82			37.49	34.04
	2.14				
AVG	**3.453333333**	Cell#4-2		23.73	20.28
	**3.45**			18.89	15.44
				44.06	40.61
				20.76	17.31
				23.78	20.33
				28.2	24.75
#5	1.2	Cell#5-1		30.98	29.135
	1.29			21.97	20.125
	1			26.98	25.135
	4.22			37.55	35.705
	1.8			18.28	16.435
	1.56			28.15	26.305
AVG	**1.845**				
		Cell#5-2		28.39	26.545
				28.51	26.665
				24.94	23.095
				17.6	15.755
				34.78	32.935
				20.46	18.615
#6	1.63	Cell#6-1		36.51	34.778
	1.46			23.29	21.558
	1.03			33.11	31.378
	1.51		tip-48.47		46.738
	3.03			29.63	27.898
AVG	**1.732**			28.32	26.588
#7	1.78	Cell#7-1		22.08	20.6
	1.32			22.03	20.55
	1.39		tip-47.92		
	1.42			18.63	17.15
	1.35			24.74	46.44
	1.64			37.89	36.41
AVG	**1.483333333**				
		Cell#7-2		40.62	39.14
				34.5	33.02
				23.78	22.3
				24.86	23.38
				24.63	23.15
				31.95	30.47
#8	0.99	Cell#8-1		20.91	19.505
	1.06			21.96	20.555
	1.01			24.16	22.755
	1.18		tip-51.06		49.655
	0.76			25.64	24.235
	3.43			20.5	19.095
AVG	**1.405**		tip-56.27		54.865
#9	1.46	Cell#9-1		22.66	21.565
	1			24.87	23.775
	1.19			30.31	29.215
	1.21			34.24	33.145
	0.84			32.61	31.515
	0.87		tip-60.11		59.015
AVG	**1.095**			32.74	31.645
				**AVE**	**25.76593939**
					n=16 cells

HA-α2C-AR/Rap1A-CA

	**BKG**			**Red Channel-BKG AVG**	**Corrected**	
#1	2.37	Cell#1-1		80.23	78	
	1.31			70.49	68.26	
	2.27			80.57	78.34	
	1.41			67.84	65.61	
	3.28			62.68	60.45	
	2.75			81.97	79.74	
AVG	**2.231666667**					
		Cell#1-2		90.81	88.58	
				96.45	94.22	
				89.59	87.36	
				95.75	93.52	
				96.11	93.88	
				94.04	91.81	
				87.11	84.88	
#2	2.6	Cell#2-1-2		70.14	67.09	
	3.8			72.2	69.15	
	3.27			77.14	74.09	
	2.71			67.9	64.85	
	3.13			71.05	68	
	2.79			77.49	74.44	
AVG	**3.05**					
		Cell#2-3		95.96	92.91	
				101.57	98.52	
				100.88	97.83	
				98.03	94.98	
				100.71	97.66	
				100.59	97.54	
		Cell#2-4		97.05	94	
				97.76	94.71	
				99.58	96.53	
				93.1	90.05	
				103.87	100.82	
				99.15	96.1	
		Cell#2-5-6		75.71	72.66	
				89.78	86.73	
				101.98	98.93	
				96.49	93.44	
				97.89	94.84	
				99.52	96.47	
#3	1.02	Cell#3-1		44.06	43.06	
	1.05			43.75	42.75	
	0.94			54.11	53.11	
	1.03			55.24	54.24	
	1.15			43.45	42.45	
	0.85			52.57	51.57	
AVG	**1.006666667**					
		Cell#3-2-3		60.78	59.78	
				67.83	66.83	
				61.39	60.39	
				56.47	55.47	
				58.36	57.36	
				49.89	48.89	
				67.99	66.99	
#4	2.42	Cell#4-1		83.18	79.96	
	2.03			73.69	70.47	
	2.76			85.93	82.71	
	3.86			93.98	90.76	
	5.29			97.32	94.1	
	2.96			82.73	79.51	
AVG	**3.22**					
		Cell#4-2		75.41	72.19	
				67.78	64.56	
				72.46	69.24	
				71.85	68.63	
				70.81	67.59	
				90.52	87.3	
		Cell#4-3		96.02	92.8	
				81.93	78.71	
				80.69	77.47	
				85.17	81.95	
				75.34	72.12	
				53.93	50.71	
		Cell#4-4		56.26-3.22	53.04	
				86.21	82.99	
				87.28	84.06	
				85.58	82.36	
				71.09	67.87	
				76.66	73.44	**77.08676**
#5	0.9	Cell#5-1		77.63	74.92	
	5.18			91.53	88.82	
	1.3			79.67	76.96	
	2.43			80.81	78.1	
	2.03			62.64	59.93	
	4.44			60.36	57.65	
AVG	**2.713333333**					
#6	1.57	Cell#6-1-2		82.62	81.14	
	0.73			95.97	94.49	
	1.66			93.88	92.4	
	3.84			87.23	85.75	
	0.38			71.92	70.44	
	0.68			78.53	77.05	
AVG	**1.476666667**					
	**1.48**					
#7	1.64	Cell#7-1		67.95	66.32	
	1.34			82.87	81.24	
	1.64			63.62	61.99	
	2.08			67.24	65.61	
	1.81			81.58	79.95	
	1.26			69.86	68.23	
AVG	**1.628333333**					
	**1.63**	Cell#7-2		88.57	86.94	
				62.35	60.72	
				67.87	66.24	
				77.53	75.9	
				82.57	80.94	
				71.61	69.98	
#8	3.24	Cell#8-1		95.47	92.34	
	3.9			104.79	101.66	
	1.92			95.88	92.75	
	2.73			92.8	89.67	
	4.27			84.19	81.06	
	2.74			94.4	91.27	
AVG	**3.133333333**					
		Cell#8-2		83.14	80.01	
				71.68	68.55	
				66.02	62.89	
				68.38	65.25	
				73.71	70.58	
				81.49	78.36	
#9	0.81	Cell#9-1		74.12	72.66	
	1.12			66.38	64.92	
	2.93			60.47	59.01	
	1.68			68.78	67.32	
	1.08			65.5	64.04	
	1.15			57.41	55.95	
AVG	**1.461666667**					
#10	3.01	Cell#10-1	tip-85.91		83.11	
	4.05			63.33	60.53	
	0.99			85.06	82.26	
	2.01			81.82	79.02	
	5.25			90.62	87.82	
	1.47			85.34	82.54	
AVG	**2.796666667**					
	**2.8**	Cell#10-2	tip-85.86		83.06	
				70.58	67.78	
				74.54	71.74	
				73.57	70.77	
			tip-59.31		56.51	
				72.3	69.5	
#11	1.69	Cell#11-1		65.45	63.37	
	2.67			74.47	72.39	
	1.48			67.87	65.79	
	1.61			80.91	78.83	
	1.63			52.9	50.82	
	3.39			78.68	76.6	
AVG	**2.078333333**					
	**2.08**	Cell#11-2		80.64	78.56	
				72.62	70.54	
				72.8	70.72	
				55.72	53.64	
				67.96	65.88	
				72.09	70.01	
		n=23 cells	**AVG**		**75.54435714**	

HA-α2C-2A-pcDNA

	**BKG**			**Texas Red/Red Channel-BKG AVG**	**Corrected**
#1	0.58	#1		13.71	12.37
	0.41			12.63	11.29
	5.88			12.59	11.25
	0.41			12.05	10.71
	0.12			13.82	12.48
	0.62			17.75	16.41
AVG	**1.336667**	**1.34**			
#2	0.32	#2		34.66	33.85
	0.91			21.61	20.8
	0.33			40.43	39.62
	0.5			52.33	51.52
	1.7		tip-78.15		77.34
	1.1			69.41	68.6
AVG	**0.81**			30.33	29.52
#2b	0.65	#2b		64.01	63.52
	0.47			72.05	71.56
	0.34			78.01	77.52
	0.16			59.66	59.17
	0.85			69.21	68.72
	0.46			93.09	92.6
AVG	**0.488333**	**0.49**		68.14	67.65
				73.84	73.35
#3a	0.76	#3a		28.91	27.45
	0.18			68.67	67.21
	1.19			37.61	36.15
	2.84			33.32	31.86
	3.6			50.92	49.46
	0.16			39.78	38.32
AVG	**1.455**	**1.46**			
#3b	0.09	#3b		64.69	63.01
	0.67			56.9	55.22
	0.3			69.49	67.81
	3.1			79.34	77.66
	3.81			50.15	48.47
	2.13			50.48	48.8
AVG	**1.683333**				
#4	0.74	#4		57.87	57.6
	0.01			61.81	61.54
	0.75			60.93	60.66
	0.04			68.44	68.17
	0.05			50.33	50.06
	0.05			53.26	52.99
AVG	**0.273333**				
#6	0	#6		75.87	75.87
	0			61.28	61.28
	0			57.31	57.31
	0			65.88	65.88
	0			66.64	66.64
	0			47.77	47.77
AVG	**0**				
#8a	0.37	#8a		38.58	38.12
	0.41			82.44	81.98
	0.31			85.24	84.78
	0.3			61.42	60.96
	0.53			66.83	66.37
	0.81			55.12	54.66
AVG	**0.455**	**0.46**			
#8c	0	#8c		37.05	37.05
	0			33.79	33.79
	0			46.95	46.95
	0			41.86	41.86
	0			45.9	45.9
	0			43.67	43.67
AVG	**0**				
#9	0	#9		76.82	76.82
	0			50.91	50.91
	0			47.73	47.73
	0			50.16	50.16
	0			57.66	57.66
	0			72.48	72.48
AVG	**0**				
#10	0.02	#10		67.75	67.65
	0.13			59.13	59.03
	0.38			43.88	43.78
	0.05			57.06	56.96
	0.003			40.3	40.2
	0.02			48.44	48.34
AVG	**0.1005**				
#11	0	#11		76.46	76.33
	0.73			74.08	73.95
	0			57.95	57.82
	0			86.99	86.86
	0.01			70.49	70.36
	0.05			73.59	73.46
AVG	**0.131667**				**54.0484**
					**n=12 cells**

HA-α2C-2A-Rap1A

	**BKG**			**Texas Red/Red Channel-BKG AVG**	**Corrected**
#1	0.97	#1		17.41	16.61
	0.49			12.61	11.81
	0.2			16.87	16.07
	0.99			22.18	21.38
	0.86			22.26	21.46
	1.31			23.5	22.7
AVG	**0.803333**				
#2	0.83	#2		30.82	30.39
	0.43			30.38	29.95
	0.37			29.73	29.3
	0.16			19.99	19.56
	0.47			41.31	40.88
	0.33			34.07	33.64
AVG	**0.431667**				
#4	0.37	#4		34.27	33.92
	0.65			25.93	25.58
	0.29			24.17	23.82
	0.41			20.26	19.91
	0.26			23.27	22.92
	0.09			13.17	12.82
AVG	**0.345**	**0.35**		18.83	18.48
#6	5.34	#6		33.2	26.66
	3.3			40.93	34.39
	7.74			44.88	38.34
	6.81			29.37	22.83
	7.35			29.33	22.79
	8.7			26.5	19.96
AVG	**6.54**				
#8	0.13	Cell#8		47.71	47.53
	0.11			26.79	26.61
	0.24			11.06	10.88
	0.32			32.7	32.52
	0.1			31.18	31
	0.19			30.72	30.54
AVG	**0.181667**				
#9	0	Cell#9		43.49	43.06
	0.14			46.31	45.88
	0			35.76	35.33
	0			53.46	53.03
	1.47			27.59	27.16
	0.94			50.29	49.86
AVG	**0.425**	**0.43**			
#12	0	Cell#12		33.91	33.91
	0			30.79	30.79
	0			38.18	38.18
	0			28.01	28.01
	0			54.58	54.58
	0			39.52	39.52
AVG	**0**				
#13	0.19	Cell#13-1		15.59	15.23
	0.37			9.48	9.12
	0.4			16.81	16.45
	0.33			17.06	16.7
	0.39			19.94	19.58
	0.5			15.38	15.02
AVG	**0.363333**				
		Cell#13-2		14.71	14.35
				18.1	17.74
				23.41	23.05
				16.37	16.01
				13.57	13.21
				17.33	16.97
#14	0	Cell#14		58.08	58.08
	0		tip-42.08		42.08
	0			34.3	34.3
	0			29.78	29.78
	0			31.74	31.74
	0			29.82	29.82
AVG	**0**				**27.76705**
					**n=10 cells**

Figure 9

NIH3T3-Co-transfections
19-Sep-23
FITC
DAPI
Texas Red
60X Oil lens

	**α2C-AR-GFP-pcDNA**					
	**No Peptide**					
	**BKG**			**FITC/Green Channel-BKG AVG**	**Corrected**	
#1	5.69	Cell#1		10.49	4.97	
	5.53			9.72	4.2	
	5.23			11.05	5.53	
	5.04			14.41	8.89	
	5.35			11.16	5.64	
	6.27			11.54	6.02	
AVG	**5.518333333**	**5.52**				
#2	4.73	Cell#2		7.26	2.66	
	4.54			8.11	3.51	
	4.69			6.43	1.83	
	4.39			9.52	4.92	
	4.59			9.86	5.26	
	4.68			10.04	5.44	
AVG	**4.603333333**	**4.6**				
#3	5.63	Cell#3		11.22	5.32	
	5.75			11.55	5.65	
	5.72			11.12	5.22	
	6.04			12.75	6.85	
	6.34			9.76	3.86	
	5.88			12.69	6.79	
AVG	**5.893333333**	**5.9**				
#5	6.56	Cell#5		12.13	5.69	
	6.43			15.06	8.62	
	7.36			11.68	5.24	
	6.43			14.96	8.52	
	5.56			10.44	4	
	6.32			9.13	2.69	**5.305**
AVG	**6.443333333**	**6.44**				n=4 cells
#7	5.98	Cell#7		10.26	4.26	
	5.83			11.92	5.92	
	5.99			15.34	9.34	
	5.72			14.14	8.14	
	6.32			13.14	7.14	
	6.21			13.89	7.89	
AVG	**6.008333333**					
#10	6.18	Cell#10		11.02	4.92	
	6.15			15.61	9.51	
	6.08			14.65	8.55	
	6.16			15.68	9.58	
	5.92			12.86	6.76	
	6.15			10.64	4.54	
AVG	**6.106666667**					
#11	6.77	Cell#11		12.6	5.91	
	6.64			15.18	8.49	
	6.93			14.3	7.61	
	6.72			15.38	8.69	
	6.25			12.74	6.05	
	6.84			13.34	6.65	
AVG	**6.691666667**	**6.69**				
#16	5.13	Cell#16	Tip-10.5		5.57	
	4.79			11.11	6.18	
	5.1			15.03	10.1	
	4.81			10.93	6	
	4.9			11.55	6.62	
	4.86			13.38	8.45	
AVG	**4.931666667**					
#19	6.98	Cell#19		22.5	15.62	
	7.14			19.64	12.76	
	6.84			13.79	6.91	
	6.75			15.4	8.52	
	6.88			17.2	10.32	
	6.69			15.45	8.57	
AVG	**6.88**					
#22	1.05	Cell#22		16.86	15.78	
	1.14			14.42	13.34	
	1.13			16.15	15.07	
	0.97			11.59	10.51	
	1.11			14.68	13.6	
	1.08			7.78	6.7	
AVG	**1.08**					
#9	6.22	Cell#9		11.99	5.95	
	6.33			12.14	6.1	
	5.89			12.78	6.74	
	5.94			19.29	13.25	
	5.76			14.79	8.75	
	6.08			15.79	9.75	
AVG	**6.036666667**	**6.04**			**7.40045455**	n=11 cells

	**α2C-AR-GFP-Rap1A-CA**				
	**No Peptide**				
	**BKG**		**FITC/Green Channel-BKG AVG**	**Corrected**	
#1	5.61	Cell#1	18.8	14.02	
	4.52		17.4	12.62	
	4.62		17.54	12.76	
	4.51		24.5	19.72	
	4.61		23.61	18.83	
	4.83		23.79	19.01	
AVG	**4.783333333**				
#3	6.25	Cell#3	19.25	13.41	
	5.78		25.22	19.38	
	5.95		23.1	17.26	
	5.72		16.97	11.13	
	5.76		15.69	9.85	
	5.6		14.25	8.41	
AVG	**5.843333333**				
#6	8.88	Cell#6	16.94	7.91	
	8.91		17.75	8.72	
	9.28		18.93	9.9	
	9.25		18.37	9.34	
	8.91		18.01	8.98	
	8.95		20.43	11.4	
AVG	**9.03**				
#9	6.7	Cell#9-1	13.66	7.41	
	6.11		11.87	5.62	
	6.19		14.32	8.07	
	5.98		13.2	6.95	
	6.1		13.07	6.82	
	6.4		12.48	6.23	
AVG	**6.246666667**				
	**6.25**	Cell#9-2	16.22	9.97	
			16.52	10.27	
			15.38	9.13	
			17.34	11.09	
			13.14	6.89	
			15.91	9.66	
#10	7.48	Cell#10	21.31	14.13	
	6.9		20.86	13.68	
	6.53		20.31	13.13	
	7.12		21.53	14.35	
	7.66		22.7	15.52	
	7.39		20.68	13.5	
AVG	**7.18**				
#11	9.48	Cell#11	28.81	19.25	
	9.58		27.4	17.84	
	9.74		27.43	17.87	
	9.67		23.1	13.54	
	9.37		25.02	15.46	
	9.5		25.68	16.12	**12.26548**
AVG	**9.556666667**	**9.56**			**n=7 cells**
#12	10.37	Cell#12	18.14	7.99	
	10.15		18.47	8.32	
	9.99		20.65	10.5	
	10.58		24.5	14.35	
	9.93		23.77	13.62	
	9.86		27.01	16.86	
AVG	**10.14666667**	**10.15**			
#16	10.65	Cell#16	25.71	15.41	
	10.07		26.69	16.39	
	9.73		29.9	19.6	
	10.87		24.7	14.4	
	10.3		29.2	18.9	
	10.2		25.99	15.69	
AVG	**10.30333333**				
#17	7.43	Cell#17	23.02	16.07	
	7.19		19.11	12.16	
	6.73		23.63	16.68	
	6.58		24.56	17.61	
	6.79		26.13	19.18	
	6.97		20.3	13.35	
AVG	**6.948333333**	**6.95**			
#18	4.49	Cell#18	27.64	23.11	
	4.41		25.57	21.04	
	4.51		25.14	20.61	
	4.56		23.35	18.82	
	4.65		22.64	18.11	
	4.56		20.76	16.23	
AVG	**4.53**			**13.6386364**	**n=11 cells**

	**α2C-AR-GFP-Rap1A-CA**				
	**Peptide A (25µl)**				
	**BKG**		**FITC/Green Channel-BKG AVG**	**Corrected**	
#1	11.21	Cell#1	22.04	11	
	10.97		26.84	15.8	
	11.2		30.95	19.91	
	10.79		27.77	16.73	
	11.05		25.07	14.03	
	11.04		24.15	13.11	
AVG	**11.04333333**				
#2	6.68	Cell#2	16.77	9.965	
	6.82		19.19	12.385	
	6.92		16.58	9.775	
	6.74		18.1	11.295	
	7		16.8	9.995	
	6.67		14.64	7.835	
AVG	**6.805**				
#4	7.76	Cell#4	18.12	10.44	
	7.69		16.26	8.58	
	7.9		16.03	8.35	
	7.63		15.99	8.31	
	7.57		19.52	11.84	
	7.55		16.11	8.43	
AVG	**7.683333333**	**7.68**	19.85	12.17	
#5	7.82	Cell#5	15.7	8.22	
	7.52		19.33	11.85	
	7.75		19.44	11.96	
	7.47		17.6	10.12	
	7.23		17.26	9.78	
	7.11		18.41	10.93	
AVG	**7.483333333**	**7.48**			
#6	10.19	Cell#6	26.79	16.53	
	9.93		26.53	16.27	
	10.22		26.74	16.48	
	10.39		25.15	14.89	
	10.57		22.96	12.7	
	10.25		23.69	13.43	
AVG	**10.25833333**	**10.26**			
#7	6.88	Cell#7	38.81	31.6	
	7.16		30.29	23.08	
	7.29		27.64	20.43	
	6.94		29.92	22.71	
	7.42		33.82	26.61	
	7.55		35.93	28.72	
AVG	**7.206666667**	**7.21**			
#8	5.86	Cell#8	34.85	28.82	
	5.91		32.06	26.03	
	5.79		17.83	11.8	
	6.18		18.45	12.42	
	6.52		19.05	13.02	
	5.89		17.46	11.43	**14.64605**
AVG	**6.025**	**6.03**			n=7 cells
#9	8.03	Cell#9	19.76	12.17	
	7.65		19.93	12.34	
	7.49		20.49	12.9	
	7.17		19	11.41	
	7.12		21.88	14.29	
	8.08		22.93	15.34	
AVG	**7.59**				
#10	8.09	Cell#10	18.65	10.31	
	8.36		17.5	9.16	
	8.27		20.64	12.3	
	8.39		23.82	15.48	
	8.53		19.59	11.25	
	8.42		20.4	12.06	
AVG	**8.343333333**				
#13	3.48	Cell#13	18.79	15.56	
	3.19		20.42	17.19	
	3.17		27.09	23.86	
	3.25		28.4	25.17	
	3.17		27.23	24	
	3.1		24.86	21.63	
AVG	**3.226666667**	**3.23**			
#16	8.41	Cell#16	18.32	10.22	
	7.94		21.96	13.86	
	8.14		26.61	18.51	
	8.4		27.79	19.69	
	7.85		26.02	17.92	
	7.87		23.61	15.51	
AVG	**8.101666667**				
#17	4.18	Cell#17	9.57	5.53	
	4.01		10.72	6.68	
	4.2		15.3	11.26	
	4.02		14.59	10.55	
	3.9		16.32	12.28	
	3.92		13.58	9.54	
AVG	**4.038333333**	**4.04**		**14.48972603**	
				**n=12 cells**	

	**α2C-AR-GFP-Rap1A-CA**				
	**Peptide C (25µl)**				
	**BKG**		**FITC/Green Channel-BKG AVG**	**Corrected**	
#1	5.48	Cell#1	10.23	4.74	
	5.53		10.99	5.5	
	5.34		10.27	4.78	
	5.7		11.16	5.67	
	5.53		11.28	5.79	
	5.35		13.12	7.63	
AVG	**5.488333333**	**5.49**			
#2	6.18	Cell#2	7.75	1.55	
	5.94		8.62	2.42	
	6.29		8.3	2.1	
	6.45		8.64	2.44	
	6.11		10.86	4.66	
	6.2		9.63	3.43	
AVG	**6.195**	**6.2**			
#4	7.48	Cell#4	10.16	2.68	
	7.49		11.21	3.73	
	7.19		14.15	6.67	
	8.06		13.84	6.36	
	7.19		14.56	7.08	
	7.47		13.05	5.57	
AVG	**7.48**				
#5	7.29	Cell#5	11.93	4.96	
	6.82		12.56	5.59	
	6.73		10.9	3.93	
	6.87		10.27	3.3	
	7.15		11.43	4.46	
	6.98		13.56	6.59	
AVG	**6.973333333**				
#8	6.52	Cell#8	15.53	9.31	
	6.28		13.22	7	
	6		13.51	7.29	
	6.24		12.88	6.66	
	6.11		21.29	15.07	
	6.16		22.36	16.14	
AVG	**6.218333333**	**6.22**	13.7	7.48	
			12.57	6.35	
#9	2.97	Cell#9	6.44	3.5	
	3.05		6.24	3.3	
	2.85		8.76	5.82	
	2.89		6.68	3.74	
	2.97		5.51	2.57	
	2.9		10.07	7.13	
AVG	**2.938333333**	**2.94**	8.94	6	
#10	3.13	Cell#10	7.28	4.11	
	3		5.21	2.04	
	2.97		5.91	2.74	
	3.19		6.36	3.19	
	3.33		5.82	2.65	
	3.37		5.25	2.08	
	**3.165**	AVG: 3.17			
#14	0.83	Cell#14	2.03	1.21	
	0.76		1.94	1.12	
	0.83		1.52	0.7	
	0.91		1.77	0.95	
	0.77		1.8	0.98	
	0.82		2.25	1.43	
AVG	**0.82**				
#16	9.32	Cell#16	16.1	7.28	
	8.51		14.64	5.82	
	8.41		19.9	11.08	
	9.26		16.66	7.84	
	8.44		14.61	5.79	
	8.98		12.06	3.24	
AVG	**8.82**				
#17	5.14	Cell#17	14.21	9.08	
	5.35		14.81	9.68	
	5.02		10.54	5.41	
	5.07		15.47	10.34	
	5.09		10.27	5.14	
	5.11		10.33	5.2	
AVG	**5.13**			**5.207777778**	n=10 cells

Figure 10

Mean Fluorescence

Panel E

solvent control			8CPT-cAMP			8CPT-cAMP PLUS Peptide C		
Mean	SEM	N	Mean	SEM	N	Mean	SEM	N
43.963	6.4483	20	129.002	11.309	24	6.56042	2.8697	24

Panel F

Control (n=4)	100	100	100	100
FSK/UK14,304 (n=4)	73	41	79	58
FSK/UK14,304/Peptide C-25µl (n=4)	147	130	132	158
FSK/UK14,304/Peptide C-50µl (n=4)	128	293	141	198
